# Vision–Language–Knowledge Co-Embedding for Visual Commonsense Reasoning

**DOI:** 10.3390/s21092911

**Published:** 2021-04-21

**Authors:** JaeYun Lee, Incheol Kim

**Affiliations:** Department of Computer Science, Kyonggi University, Suwon-si 16227, Korea; wodbs9522@kyonggi.ac.kr

**Keywords:** visual commonsense reasoning, multimodal co-embedding, knowledge graph, graph convolutional network, pretrained multi-head self-attention network

## Abstract

Visual commonsense reasoning is an intelligent task performed to decide the most appropriate answer to a question while providing the rationale or reason for the answer when an image, a natural language question, and candidate responses are given. For effective visual commonsense reasoning, both the knowledge acquisition problem and the multimodal alignment problem need to be solved. Therefore, we propose a novel Vision–Language–Knowledge Co-embedding (ViLaKC) model that extracts knowledge graphs relevant to the question from an external knowledge base, ConceptNet, and uses them together with the input image to answer the question. The proposed model uses a pretrained vision–language–knowledge embedding module, which co-embeds multimodal data including images, natural language texts, and knowledge graphs into a single feature vector. To reflect the structural information of the knowledge graph, the proposed model uses the graph convolutional neural network layer to embed the knowledge graph first and then uses multi-head self-attention layers to co-embed it with the image and natural language question. The effectiveness and performance of the proposed model are experimentally validated using the VCR v1.0 benchmark dataset.

## 1. Introduction

**Figure 1:**
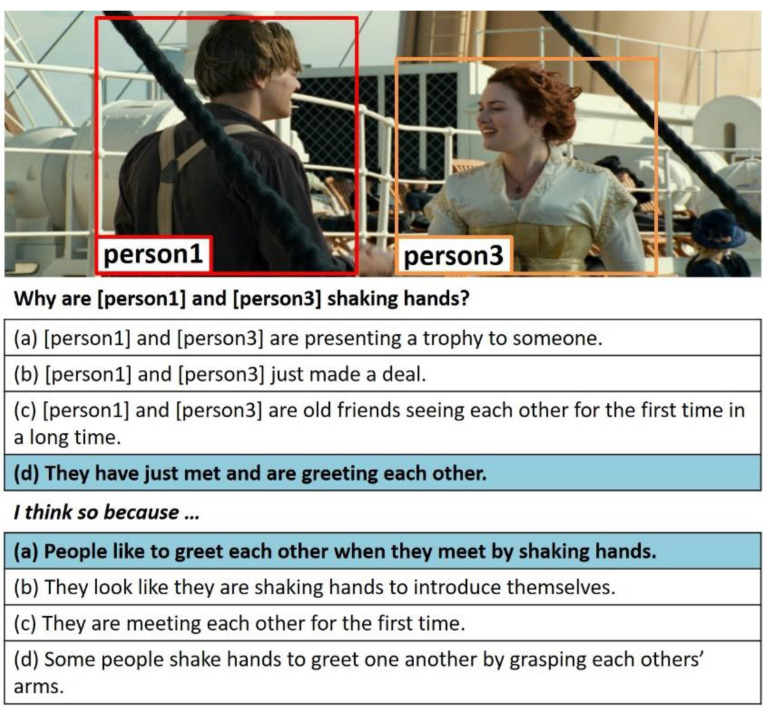
Examples of visual commonsense reasoning (VCR) tasks.

Nowadays, there is increasing interest in HCI research problems requiring both visual and linguistic understanding such as visual question-answering (VQA) [1], visual commonsense reasoning (VCR) [2], vision-and-language navigation (VLN) [3,4,5,6], and visual dialog (VD) [7]. Techniques for solving these problems can be used for various useful applications: intelligent personal assistants, chatbots, and robots. Visual commonsense reasoning (VCR) [2] is an intelligent task performed to decide the most appropriate answer to a question while providing the rationale or reason for the answer when an image, a natural language question, and candidate responses are given, as shown in Figure 1. Unlike conventional visual question-answering (VQA) [1], VCR requires additional commonsense reasoning for the social relationships between the persons in the image and natural language questions and the causal relationships between the events. Therefore, the following problems must be solved to effectively perform VCR tasks.

The first is the knowledge acquisition problem, which concerns obtaining the commonsense knowledge needed to answer a given question. Most past VCR models tried to acquire this commonsense knowledge only from the VCR v1.0 training dataset through learning without using external knowledge sources. High-quality commonsense knowledge will be obtained more effectively if relevant knowledge is retrieved from an external well-defined knowledge base and used to train the model along with the VCR training dataset. However, integrating the external knowledge of a graphical structure or multiple (subject, predicate, object) triples with the RGB image and the natural language question of different modalities can be a problem.

The second is the multimodal alignment problem, which concerns correlating different modal data such as the RGB image and the natural language question to understand interrelationships between them. In recent years, convolutional neural networks (CNNs) [8] have been successfully used to obtain rich feature maps of two-dimensional (2D) images in the field of computer vision. In contrast, recurrent neural networks (RNNs) [9] have been used to represent natural language sentences, which are sequences of words, in the natural language processing field. Since then, pretrained language models, such as BERT [10] and GPT [11], have been commonly used for natural language embedding. In general, pretrained language models use a multi-head self-attention network or Transformer to embed natural language sentences. Such pretrained language models have an advantage in that their multi-head self-attention network can better facilitate parallel processing than RNN. Furthermore, such language models can better reflect the semantics and structural context information of text by being pretrained with several large corpuses such as Wikipedia.

In early studies on VCR, models using an RNN and the attention mechanism were often proposed to embed a natural language question while aligning with the corresponding input image [2,12,13]. Recently, some pretrained self-attention models such as ViLBERT [14], VisualBERT [15], Unicoder-VL [16], B2T2 [17], and CBM [18] have emerged, which can co-embed a natural language text and an image together. However, these models do not provide an effective method to use an external knowledge graph required for the VCR task, together with the input image and the natural language text.

This paper proposes a novel model, Vision–Language–Knowledge Co-embedding (ViLaKC), to address these problems of VCR task. This model extracts knowledge graphs relevant to the given question and the input image from an external knowledge base, ConceptNet [19], and then uses them together with the input image and the natural language question in response reasoning. Furthermore, the proposed model uses a pretrained vision–language–knowledge embedding module, which embeds three different modal data, such as images, natural language questions, and knowledge graphs, into a single multimodal feature vector. In particular, the proposed model embeds a knowledge graph into a knowledge vector using a graph convolutional neural network layer (GCN) [20] and then co-embeds it with the visual and the textual vectors using multi-head self-attention layers. Furthermore, several tasks including (1) masked language modeling with image and knowledge (MLMIK), (2) masked object classification with text and knowledge (MOCTK), and (3) vision–language–knowledge matching (VLKM), are proposed to pretrain the vision–language–knowledge embedding module. This study validates the excellence of the proposed model through various experiments using VCR v1.0, a benchmark dataset.

The contributions of this paper are summarized as follows.

- ViLaKC, a novel VCR model, is proposed, which uses an external knowledge base to solve the commonsense knowledge acquisition problem.
- This study proposes a method that extracts the relevant knowledge from the ConceptNet knowledge base and then effectively embeds the retrieved knowledge graph using GCN.
- A pretrained self-attention network-based vision–language–knowledge embedding module is proposed to integrate multimodal data efficiently.
- Several pretraining tasks have been proposed for reflecting interrelationships between images, languages, and knowledge.

In Section 2 of this paper, related works are briefly examined, and in Section 3 detailed design issues of the proposed ViLaKC model are introduced. In Section 4, the implementation of the proposed model and the results of various experiments are explained, and in Section 5 the conclusions are summarized.

## 2. Related Works

### 2.1. Visual Commonsense Reasoning

Some early studies on VCR proposed models that combined an RNN and attention mechanism to align between the input image and the natural language question. In the R2C [2] model, the image and the natural language question are embedded independently using Mask R-CNN [21] and BERT [10], respectively. These two feature vectors are mutually aligned using abilinear attention network (BAN) [22], and an RNN, long short-term memory (LSTM). Unlike the R2C [2] model, TAB-VCR [12] performs joint encoding of the visual and textual vectors using LSTM-RNN, instead of complex BAN. The simpler structure of the TAB-VCR can reduce the number of learning parameters to lower the model complexity. Further, TAB-VCR also achieved significant improvement over the original VCR v1.0 dataset by adding various attribute tags of each object in the input image and enhancing the quality of image-text grounding. Therefore, TAB-VCR is a much simpler model than the R2C baseline model, but it achieves significantly better results. However, both the R2C and the TAB-VCR models cannot produce interpretable reasoning paths or solely explore intra-relationship of visual objects while ignoring the cross-domain semantic alignment among visual concepts and linguistic words. A new Heterogeneous Graph Learning (HGL) model [13] integrates seamlessly the intra-graph and inter-graph reasoning in order to semantically align between vision and language domains. Moreover, to interactively refine reasoning paths for semantic agreement, HGL includes a primal vision-to-answer heterogeneous graph (VAHG) module and a dual question-to-answer heterogeneous graph (QAHG) module.

In recent years, there has been a rapid proliferation of studies using a pretrained multi-head self-attention network. Recent pretrained multi-head self-attention networks that can embed a vision and natural language together include ViLBERT [14], VisualBERT [15], Unicoder-VL [16], B2T2 [17], and CBM [18]. ViLBERT [14], VisualBERT [15], and Unicoder-VL [16] are VCR task-agnostic models. ViLBERT [14] has a two-stream architecture and embeds image regions and text segments independently through transformer blocks and co-attentional transformer layers, respectively. The contextualized representations between vision and language are obtained through the co-attentional transformer layers placed in the middle. In contrast, VisualBERT [15] and Unicoder-VL [16] have a single-stream architecture in which image regions and text segments are co-embedded through the same transformer blocks. It is generally known that single-stream models such as VisualBERT and Unicoder-VL outperform two-stream models such as ViLBERT while being significantly simpler. Single-stream models avoid making changes to the BERT architecture, resulting in significantly deeper visual processing and earlier interaction between modalities. 

Both B2T2 [17] and CBM [18] are VCR task-specific models. B2T2 (Bounding Boxes in Text Transformer) [17] is an early fusion single-stream architecture where visual features are embedded on the same level as input word tokens. Visual features of bounding boxes are inserted where they are mentioned in the text and at the end of the input. Therefore, text, image, and bounding boxes are combined at the level of the non-contextualized token representations rather than right before the classification decision. CBM (Curriculum-based Masking) [18], another single-stream model, addresses the issue that insignificant changes may cause VCR models’ performance to degrade significantly. This model uses a curriculum-based masking as a mechanism to perform more robust training. It requires one to pay attention to the answers as a whole and is more effective than prior masking strategies. 

The tasks performed to pretrain them include the masked language modeling (MLM) task, masked object classification (MOC) task, and visual-linguistic matching (VLM) task. Among them, the MLM and VLM tasks are used in the pretraining of ViLBERT, VisualBERT, and Unicoder-VL networks, while the MOC task is used in the pretraining of Unicoder-VL only. Furthermore, these multi-head self-attention networks require a large dataset composed of image-text pairs for pretraining. For example, a Conceptual Captions dataset is used for the pretraining of the ViLBERT, MSCOCO Captions dataset for VisualBERT, and Conceptual Captions and SBU Captions datasets for the Unicoder-VL.

Pretrained multi-head self-attention models such as ViLBERT, VisualBERT, and Unicoder-VL have been applied to various tasks such as VQA, visual dialog, and VCR, thereby proving their effectiveness in mutually aligning images and natural language texts. However, the VCR task discussed in this study requires the use of the external knowledge graphs in addition to the input images and natural language questions. These pretrained self-attention models, ViLBERT, VisualBERT, and Unicoder-VL, cannot co-embed knowledge graphs together with images and natural language texts. Therefore, a new self-attention model that can co-embed three different modal data in an efficient manner is desired.

### 2.2. External Knowledge Base

Unlike conventional VAQ tasks, VCR requires additional commonsense reasoning to find social relationships between persons or causal relationships between events appearing in the input image and/or the question. Most previous VCR models acquired commonsense knowledge only through learning with the VCR dataset or the additional image-text dataset. However, it is not easy to learn a set of broad commonsense knowledge from a small VCR dataset or a large unstructured image-text dataset from the scratch. It would be more efficient and effective to use a part of commonsense knowledge relevant to the given VCR task retrieved from an external well-defined knowledge base.

There are several large-scale open knowledge bases such as ConceptNet [19], Freebase [23], DBPedia [24], and WikiData [25]. Among them, ConceptNet contains a broad range of conceptual commonsense knowledge, such as a hierarchy of entity classes, a set of entity attributes, and possible relationship types between entity classes. In contrast, FreeBase, DBpedia, and WikiData contain mostly factual knowledge of the real world, such as “Barack Obama is a politician”.

An important concern when designing a VCR model using external knowledge is how to embed the retrieved knowledge into a feature vector. Most external knowledge forms a graphical structure or a set of (subject, predicate, object) triples. Therefore, a graph embedding method that is different from visual or textual embedding methods is needed. There are some graph embedding methods such as DeepWalk [26], node2vec [27], AttentionWalk [28], and GCN [20]. Among them, GCN [20] is a well-known graph neural network (GNN) that can embed nodes of a graph efficiently by exchanging rich context information between neighboring nodes.

Some existing studies on natural language processing (NLP) have proposed pretrained self-attention network models such as KnowBERT [29] and C2T [30] to embed a natural language text with its associated knowledge. However, such a language-knowledge co-embedding model as-is cannot be applied in VCR tasks that require both visual and language understanding. Therefore, these models need to be further extended to embed visual information together for VCR tasks.

To the best of our knowledge, no VCR model that can use an external knowledge base has been reported yet. However, some studies have attempted to enhance conventional VQA tasks to use external knowledge bases. In the original version of the VQA dataset v1.0 released in 2016, most of the questions could be answered immediately by simply performing proper analyses of the images or questions such as “how many people are there in the image?” There was no need to use separate external knowledge or perform deep commonsense reasoning to answer these questions. To overcome the limitations of the VQA dataset v1.0, new VQA datasets such as FVQA [31,32], KVQA [33], and OK-VQA [34] have emerged. These all provide the external knowledge required for answer inference in addition to the input image and the natural language question. FVQA provides the conceptual commonsense knowledge extracted from knowledge bases such as DBpedia [24] and ConceptNet [19] with question–answer pairs such as (Q: Which animal in this image is able to climb trees? A: Cat). In contrast, KVQA provides a variety of world knowledge extracted from the knowledge base of WikiData [25] with question–answer pairs such as (Q: Who is to the left of Barack Obama? A: Richard Cordray). Meanwhile, OK-VQA provides the documents extracted from the Wikipedia corpus with question–answer pairs such as (Q: Which American president is associated with the stuffed animal seen here? A: Teddy Roosevelt). These datasets, which are extended versions of the original VQA v1.0, are partially related to the VCR v1.0 dataset in that deep reasoning using external knowledge resources is required to answer the questions. However, these datasets differ vastly from the VCR v1.0 dataset in that not only is an answer to the question required, but also a rationale or reason, and conceptual commonsense reasoning is crucially required for most questions.

## 3. Visual Commonsense Reasoning Model

### 3.1. Model Overview

In this paper, we propose a novel deep neural network model for VCR [2] tasks. The VCR tasks can be presented in three modes as follows:

- Q → A: The task of selecting an answer *a* for question *q*.
- QA → R: The task of selecting a correct rationale *r* for question *q* and answer *a*.
- Q → AR: The task of selecting an answer *a* and a correct rationale *r* for question *q*.

It is assumed that the following inputs are provided for each VCR task:

- An image I.
- A sequence of object detections v.
- A question q, in which a natural language text and pointing tags are mixed.
- Candidate responses r, in which natural language texts and pointing tags are mixed.

The input image I is provided with a set $v\;=\;\left\{\left(o_{1}.Label\left(o_{1}\right)\right),\left(o_{2}.Label\left(o_{2}\right)\right),\ldots ,\left(o_{i}.Label\left(o_{i}\right)\right)\right\}$, where $o_{i}$ indicates each object area detected in the image I and $Label\left(o_{i}\right)$ is the corresponding object’s label. The question q and candidate responses r include pointing tags such as <person 3> that indicate persons and objects appearing in the image in addition to a natural language text such as (Q: Why are [person 1] and [person 3] shaking hands?) Four candidate responses are provided for each q, assuming that exactly one response is correct.

As explained earlier, the commonsense knowledge acquisition problem and multimodal alignment problem must be resolved to perform a VCR task. For solving the commonsense knowledge acquisition problem, this study proposes a novel ViLaKC model, which extracts relevant knowledge from an external knowledge base ConceptNet and uses it with the input image and natural language question in the answer inference. Furthermore, to achieve contextualized embedding between the image, natural language text, and knowledge graph, having data of different modalities, the ViLaKC model includes a vision–language–knowledge embedding module (VLKEM).

**Figure 2:**
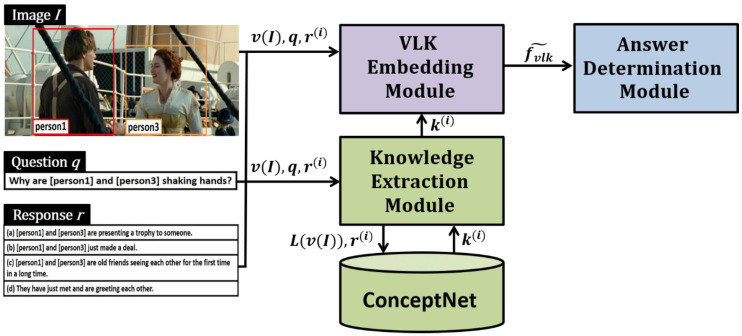
Overall structure of the proposed ViLaKC model.

As shown in Figure 2, the proposed ViLaKC model consists of the following modules: (1) knowledge extraction module (KEM), (2) vision–language–knowledge embedding module (VLKEM), and (3) answer determination module (ADM). The KEM extracts search keywords from the input image I, natural language question q, and candidate responses $r^{\left(i\right)}$ and obtains related knowledge $k^{\left(i\right)}$ from an external knowledge base, ConceptNet. The VLKEM co-embeds the image, natural language texts (question and candidate responses), and external knowledge through two stages into a multimodal co-embedding vector $\tilde{f_{VLK}}$. In the first stage, each piece of data is embedded independently by reflecting their unique characteristics: (1) the image is embedded using ResNet101, a pretrained convolutional neural network (CNN); (2) the natural language question and the candidate responses are embedded using BERT, a pretrained language model; and (3) the external knowledge of a graph structure is embedded using a graph convolutional network (GCN). The second stage of the VLKEM is a co-embedding stage in which the independently embedded multimodal feature vectors are integrated into one using a multi-head self-attention-based co-embedder pretrained with the VCR v1.0 dataset. The ADM evaluates the candidate responses based on the co-embedded rich multimodal feature information and chooses a response with the highest evaluation score as the answer. Thus, the novel ViLaKC model has the advantage of utilizing a well-defined external knowledge base in determining answers, unlike the conventional VCR models, which extract the knowledge required for determining the answer from only the input image, natural language question, and candidate responses. In addition, the multi-head self-attention network-based VLKEM achieves mutual alignment between input data of different modalities.

### 3.2. Knowledge Extraction

**Figure 3:**
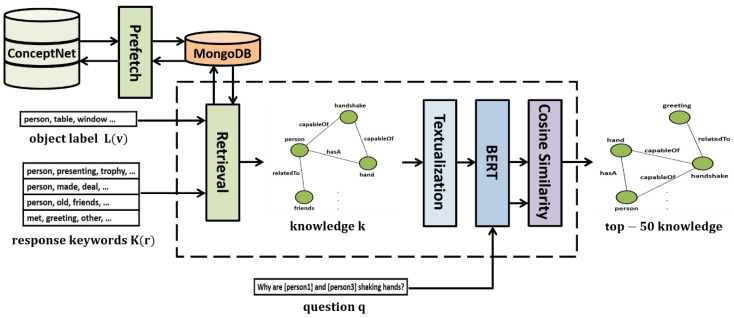
Knowledge extraction module (KEM).

Extracting the relevant knowledge needed in the answer determination effectively from ConceptNet [19] is an important design problem of the ViLaKC model. ConceptNet contains conceptual knowledge in a format of about 34 million triples (subject, predicate, object), predefined by experts. Considering the internet conditions, access restrictions, and real-time processing, it may be inefficient to search online for relevant knowledge directly from the ConceptNet site every time a question is asked. Therefore, as shown in Figure 3, the ViLaKC model uses as keywords both the labels of objects detected in the input images and concept words extracted from the natural language question and candidate responses in the VCR v1.0 dataset [2]. For the example in Figure 1, the object labels detected in the input image such as “person” and concept words extracted from the question and candidate responses such as “shaking”, “hand”, and “greeting” are used as keywords for prefetching relevant knowledge. This allows prefetching approximately three million data of associated knowledge in advance from the ConceptNet and stores them in a local server which are retrieved as needed. MongoDB is a highly scalable and flexible NoSQL database widely used for knowledge storage and management. Each ConceptNet knowledge of (subject, predicate, object) format is stored in MongoDB with “e1” indicating a subject, “rel” indicating a predicate, “e2” corresponding to an object, “_id” corresponding to the unique number of each knowledge, and “text” corresponding to a simple sentence that represents the corresponding triple.

Based on the prefetched knowledge base in MongoDB, the KEM module performs the following procedures whenever a new question q is given, as shown in Figure 3. Using the words K(r) extracted from the natural language question q, candidate responses r, and the labels $L\left(v\right)\;=\;\left\{Label\left(o_{1}\right),\;Label\left(o_{2}\right),\;\ldots ,\;Label\left(o_{k}\right)\right\}$ of the objects detected in the image as keywords, the module searches 100 relevant knowledge triples for each keyword from the MongoDB. The retrieved knowledge triples are then converted into simple sentences through textualization to calculate the similarity with the natural language question q. The natural language question q and a knowledge sentence k are embedded using the BERT [10], and the cosine similarity is calculated between the two using Equation (1):

(1) $$cosine\left(q,k_{i}\right)\;=\;\frac{q\cdot k_{i}}{\left|\left|q\right|{|}_{2}\right|\left|k_{i}\right|{|}_{2}},$$

Based on this method, the KEM extracts the top 50 associated knowledge data with the highest similarities for question q.

### 3.3. Multimodal Embedding

The VLKEM module is used to integrate and embed the input image, natural language question, and candidate responses, and relevant knowledge graph extracted from ConceptNet into a single vector. When integrating and embedding multimodal data such as image, language, and knowledge for a VCR task, it is important to represent embedding between different data while maintaining the unique characteristics of each piece of data. Therefore, in this study, the VLKEM process was divided into two main stages. In the first stage, the image, language, and knowledge are embedded independently by applying the most suitable method for each data type. The input image is embedded using ResNet. The question and candidate responses expressed in a natural language are embedded using a pretrained BERT, and the knowledge graph is embedded using GCN, which is effective for graph embedding. In the second stage, the independently embedded data are co-embedded using a pretrained self-attention network to effectively align the data into a single vector.

**Figure 4:**
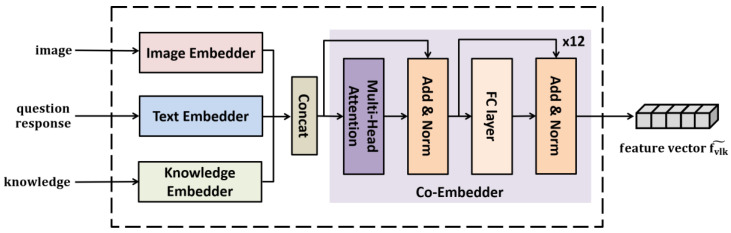
Vision–Language–Knowledge embedding module (VLKEM).

Figure 4 shows the internal configuration of the VLKEM. This module comprises an Image Embedder, a Text Embedder, and a Knowledge Embedder to independently embed the image, natural language texts, and knowledge, respectively, and a Co-embedder, which integrates the feature vectors of different modalities. 

**Figure 5:**
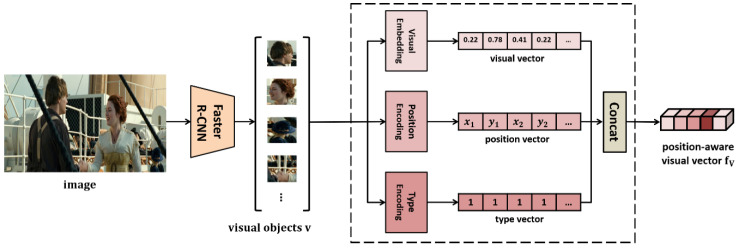
Image embedder.

Image embedding is the process of integrating the generated visual, position, and type vectors. The structure of the Image Embedder is shown in Figure 5. 

The object detector, Faster R-CNN [35], detects each object region in the input image. ResNet [36] extracts a visual vector from each object region. The position vector is obtained based on the position and size of the bounding box showing each object region using Equation (2).

(2) $$position\;vector\;=\;\left[x_{1},\;y_{1},\;x_{2},\;y_{2},\;w,\;h,\;w*h\right],$$

where $\left(x_{1},\;y_{1}\right)$ and $\left(x_{2},\;y_{2}\right)$ refer to the upper-left and bottom-right coordinates, respectively, and w and h denote the width and height of the image, respectively. Furthermore, to distinguish the image modality from the natural language and knowledge modalities, a type vector filled with 1 is generated. These three vectors are concatenated to create a position-aware visual vector $f_{V}$.

**Figure 6:**
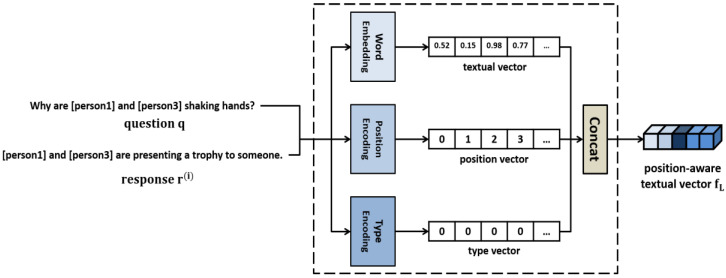
Text embedder.

As shown in Figure 6, the text embedder generates a textual vector, a position vector, and a type vector from the natural language question and candidate responses, and concatenates them into one. The text vector is obtained by embedding the question and candidate responses using a pretrained language model BERT [10], and the position vector is generated based on the position information of each word. Furthermore, a text-type vector is set to distinguish it from the image and knowledge. The text vectors, position vectors, and type vectors generated are concatenated to obtain a position-aware textual vector $f_{L}$.

**Figure 7:**
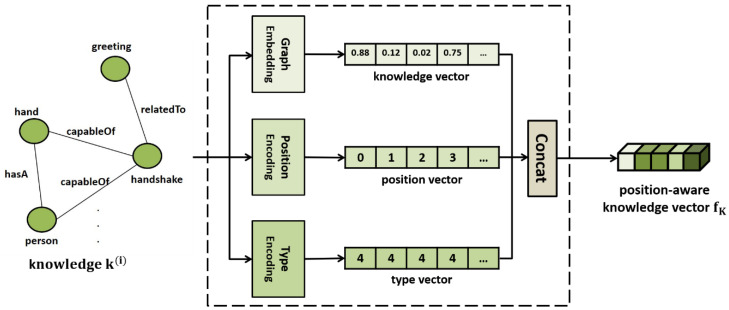
Knowledge embedder.

As shown in Figure 7, the knowledge embedder receives the retrieved knowledge graph as input and embeds it into a vector. A knowledge graph comprises nodes that represent entities or concepts and edges that represent the relationships between them. It is important for the knowledge embedder to accurately reflect the relationships between the entities in the knowledge graph. There are several graph embedding methods, such as DeepWalk [26], node2vec [27], and AttentionWalk [28], but the proposed model uses GCN [20], which can most effectively reflect the context information between the neighboring nodes. The GCN updates the information of each entity node of a knowledge graph by gathering the information of neighbor nodes connected by edges, as shown in Equation (3), to include rich relationship information in entity nodes.

(3) $$f^{\left(l+1\right)}\;=\;\sigma \left(A\cdot f^{\left(l\right)}\cdot W^{\left(l\right)}\;+\;b^{\left(l\right)}\right),$$

where *A* denotes the adjacent matrix of the knowledge graph, $f^{\left(l\right)}$ denotes the feature vector value of each entity node of layer l, $W^{\left(l\right)}$ and $b^{\left(l\right)}$ denote the weight and bias of layer l, respectively. If the relationship information is sufficiently included in each entity node while passing through the GCN layer, the feature vectors of all entity nodes on the knowledge graph are concatenated into one to generate a knowledge vector. Meanwhile, the node number of each entity on the knowledge graph is gathered to generate a position vector. Furthermore, the knowledge type vector is set to distinguish it from the image and natural language. The generated knowledge vector, position vector, and type vector are concatenated to obtain a position-aware knowledge vector $f_{K}$.

As shown in Equation (4), the co-embedder performs the role of mutually aligning $f_{V},\;f_{L},\;\mathrm{and}\;f_{K}$, which are the position-aware feature vectors generated independently by the image encoder, text embedder, and knowledge encoder, respectively, and integrating them into a single multimodal feature vector $\tilde{f_{VLK}}$.

(4) $$\tilde{f_{VLK}}\;=\;FC\left(MHAN\left(f_{V},\;f_{L},\;f_{K}\right)\right)$$

where MHAN denotes the multi-head self-attention network, and FC denotes the fully connected layer. MHAN consists of 12 self-attention layers. Before using the co-embedder in the VLKEM, it underwent a pretraining process for accurate embedding between vision, language, and knowledge. 

Many training data are required to learn the countless parameters of the co-embedder. Conventional co-embedding models such as ViBERT, VisualBERT, and Unicoder-VL require a large dataset of more than 1.3 million data for pretraining. Therefore, the VCR v1.0 dataset, which is a small dataset consisting of approximately 200,000 data, is insufficient to fully train the model with all the parameters of the co-embedder, which embeds vision, language, and knowledge together. Therefore, in this study, pretraining is performed over two stages for the co-embedder. The first is the task-agnostic pretraining stage, in which a large dataset of over 1.3 million data such as COCO, Visual Genome, Conceptual Captions, and SBU Caption is used to train the contextualized representation of vision and language. The pretraining tasks performed in this stage include (1) masked language modeling with image (MLMI), (2) masked object classification with text (MOCT), and (3) vision–language-matching (VLM). The second is the task-specific pretraining stage, in which approximately 200,000 data, including the relevant knowledge extracted from the VCR v1.0 dataset and ConceptNet, are used to train the ability for contextualized embeddings between vision, language, and knowledge required in the VCR tasks. The pretraining tasks performed in this stage include (1) masked language modeling with image and knowledge (MLMIK), (2) masked object classification with text and knowledge (MOCTK), and (3) vision-language-knowledge matching (VLKM). 

MLMI involves predicting the masked words in an input text based on the surrounding words in the text and input image, and MLMIK uses the related knowledge when predicting the masked words. Therefore, the MLMIK task randomly masks 15% of the words constituting the input text and then predicts the masked words using the input image, related knowledge, and surrounding words, excluding the masked words. MOCT refers to the task of predicting the class of masked object regions in an input image based on different object regions in the same image and input text. In contrast, MOCTIK is a task that uses related knowledge when predicting the class of masked object regions. Therefore, the MOCTK task randomly selects 15% from the object regions in the input image and performs the masking by zeroing out their visual features. Subsequently, the class of masked object regions is predicted using the input text, associated knowledge, and remaining object regions after excluding the masked regions in the image. In contrast, VLM refers to a task that determines whether the object regions in the input image and words in the input text are matched correctly, and VLKM determines whether the related knowledge, and the image and text, match correctly with each other. The semantically matching image-text-knowledge triples extracted from the VCR v1.0 dataset and ConceptNet in the VLKM task were used as positive examples, and the triples, which were not, were used as negative examples in the co-embedder training. By pretraining the co-embedder through two-stage pretraining tasks, the co-embedder learns to mutually align and embed the image, natural language texts, and related knowledge, which are the input data of different modalities.

When the pretrained co-embedder generates a multimodal co-embedding vector $\tilde{f_{VLK}}$, the answer determination module (ADM) uses a fully connected layer and softmax layer based on the vector $\tilde{f_{VLK}}$ to evaluate the candidate responses and determine the final answer.

## 4. Implementation and Experiments

### 4.1. Dataset and Model Training

In this study, we conducted experiments using a benchmark dataset VCR v1.0 [2] to evaluate the performance of the proposed model. The dataset was used to extract still images from movie clips to explain complex situations that humans can decipher (e.g., the reason why [person 1] and [person 3] in Figure 1 are shaking hands). The extracted images are provided to the Amazon Mechanical Turk (AMT) along with additional sentence information in a caption format, which provides one to three questions for each image and a reasonable answer and rationale for each question. A total of 212,923 image–question–answers were used to train the model, and 26,534 image–question–answers were used to evaluate the model.

The model was implemented using Pytorch, a deep learning library of Python in Ubuntu 16.04 LTS, and a hardware device equipped with a GeForce GTX 1080 Ti GPU was used for the implementation and experiments. The batch size and epoch for the model training were set to 4000 and 20,000, respectively. Cross-entropy was used as a loss function, and AdamW was used as the optimization algorithm.

### 4.2. Evaluation of Knowledge Graph Embedding Methods

The first experiment was conducted to verify the effectiveness of the GCN-based knowledge graph embedding method employed in the ViLaKC model. In this experiment, we compared the performance of four knowledge graph embedding methods, DeepWalk [26], node2vec [27], AttentionWalk [28], and GCN [20].

The experimental results in Table 1 show that GCN [20], which is a graph neural network, displays a higher performance than random walk-based embedding methods such as DeepWalk [26], node2vec [27], and AttentionWalk [28]. Among the random walk-based embedding methods, DeepWalk [26] showed a relatively higher performance. These experimental results demonstrate the positive effect of the GCN-based knowledge graph embedding method, which can properly reflect the relationship information between the entity nodes.

### 4.3. Evaluation of Multimodel Embedding Methods

**Figure 8:**
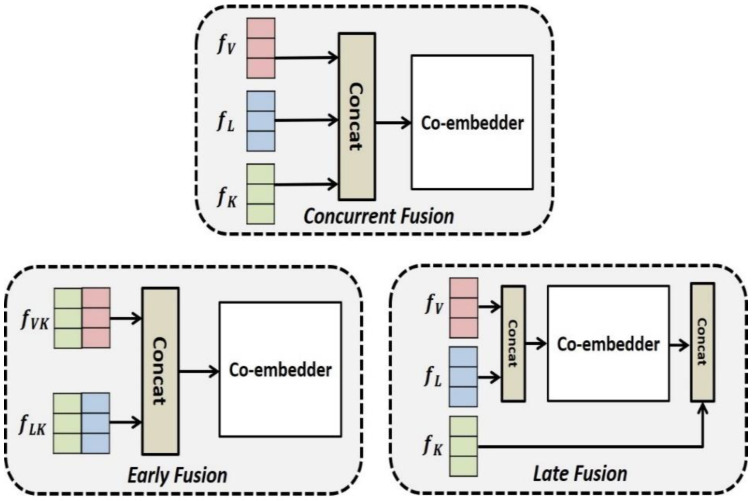
Multimodal embedding methods.

We conducted a second experiment to verify the validity of the vision–language–knowledge embedding method employed in the ViLaKC model. Figure 8 shows the three typical co-embedding vision language knowledge methods. The first is the early fusion (EF) method, which concatenates the knowledge vector $f_{K}$ with the visual vector $f_{V}$ and text vector $f_{L}$ and then embeds the two mixed feature vectors $f_{VK}$ and $f_{LK}$ through a self-attention-based co-embedder. The second is the late fusion (LF) method, which embeds $f_{V}$ and $f_{L}$ through the co-embedder and then concatenates the vision–text mixed vector $f_{VL}$ with $f_{K}$. The third is the concurrent fusion (CF) method, which co-embeds $f_{V}$, $f_{L}$, and $f_{K}$ together through the self-attention-based co-embedder, similar to the VLKEM of the ViLaKC model. In this experiment, we compared the performances of the three embedding methods.

The experimental results in Table 2 show that the CF method performs better in all Q → A, QA → R, and Q → AR compared to the EF and LF methods. In contrast, the EF method, which concatenates the related knowledge with the image and the language, shows the lowest performance compared to the LF and CF methods, which do not concatenate them first. These results confirm that it is highly effective to embed each multimodal piece of data independently using a unique embedding method suitable for the corresponding modality. Furthermore, it is confirmed that the CF method, which embeds three independently embedded multimodal features simultaneously through the self-attention-based co-embedder (like the ViLaKC model), is better than the LF method, which co-embeds the image and text first and then concatenates them with the knowledge. Based on these experimental results, we confirm the superiority of the ViLaKC model using the CF method. Unlike EF method, the LF method shows slightly lower performance than the CF method. Therefore, the LF method which has advantages such as parsimony and ease of implementation may be a good alternate for the CF method.

### 4.4. Evaluation of Pretraining Tasks

We conducted a third experiment to analyze the effects of the pretraining tasks for the co-embedder, which performs the contextualized embedding between the multimodal data. In this experiment, we performed the task-agnostic pretraining tasks of the first stage, commonly among the two pretraining stages. Then, we compared the performance between the task-specific pretraining tasks of the second stage, which used the VCR v1.0 dataset and ConceptNet’s related knowledge, as shown in Table 3. In Table 3, MLMIK+MOCTK, MLMIK+VLKM, MOCTK+VLKM, and MLMIK+MOCTK+VLKM are the respective combinations of the basic pretraining tasks used in training.

The experimental results in Table 3 show that MLMIK+MOCTK+VLKM, with all three basic tasks used for pretraining the co-embedder, similar to the ViLaKC model, performed better compared to all other cases. When we examined the cases of using two different tasks together, we found that the performance was higher in the cases of MLMIK+VLKM and MOCTK+VLKM, in which the VLKM task was used together in common, compared to MLMIK+MOCTK, which did not. Furthermore, the performance was higher in the cases of MLMIK+VLK and MOCTK+VLKM, in which the training was performed using the VLKM task together, compared to the cases of MLMIK and MOCT, in which the co-embedder was trained using only one task. In contrast, the performance was lower in MLMIK+MOCT, in which the VLKM task was not used together, compared to MLMIK, in which the training was performed using only a single task. This may be interpreted as a result of the positive effect of VLKM task when pretraining for the contextualized embeddings between the three different modalities. In contrast, when the co-embedder was pretrained using a single task VLKM, the performance was lower than other cases, meaning that the synergy effect can be obtained only when it is applied together with other tasks such as MLMIK and MOCTK. Based on these experimental results, we confirm the positive effects of the three tasks applied to the pretraining of the co-embedder in the ViLaKC model. Additionally, we also confirm the MLMIK pretraining task does much more contributions on performance than the MOCTK and VLKM tasks.

### 4.5. Comparison with Existing Models

In the fourth experiment, we compared the performance of the proposed model with the existing models, such as Chance, which chooses an arbitrary answer, three existing VCR models (R2C [2], TAB-VCR [12], and HGL [13]), and vision–language pretraining models (ViLBERT [14], VisualBERT [15], and Unicoder-VL [16]), as shown in Table 4.

The experimental results in Table 4 confirm that the proposed model shows the highest performance in terms of Q → A, QA → R, and Q → AR compared to all other models. In particular, the proposed model shows a clear performance improvement of approximately 5.1% for the Q → A type questions, about 5% for the QA → R type questions, and approximately 7.3% for the Q → AR type questions, on average, compared to the existing R2C, TAB-VCR, and HGL models. Compared to the vision–language pretraining models such as VisualBERT, ViLBERT, and Unicoder-VL, it shows an average performance improvement of approximately 0.8% for the Q → A type questions, approximately 0.9% for the QA → R type questions, and approximately 1.3% for the Q → AR type questions. The superiority and high performance of the ViLaKC model proposed in this study are confirmed based on these experimental results. Comparing with RNN-based models such as R2C, HGL, and TAB-VCR, pretrained multi-head self-attention models such as VisualBERT, ViLBERT, and Unicoder-VL show an average performance improvement of approximately 0.9–8.8% for the Q → A type questions, approximately 1.0–7.5% for the QA → R type questions, and approximately 1.8–11.3% for the Q → AR type questions. Based on these results, we also find out that pretrained multi-head self-attention models are more effective than RNN-based models for VCR tasks.

### 4.6. Qualitative Analysis

**Figure 9:**
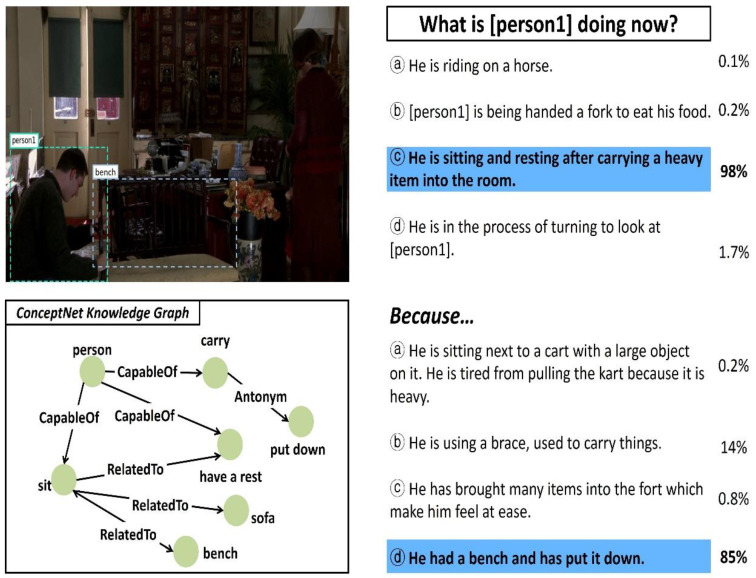
The first example of VCR task performed by the proposed model.

**Figure 10:**
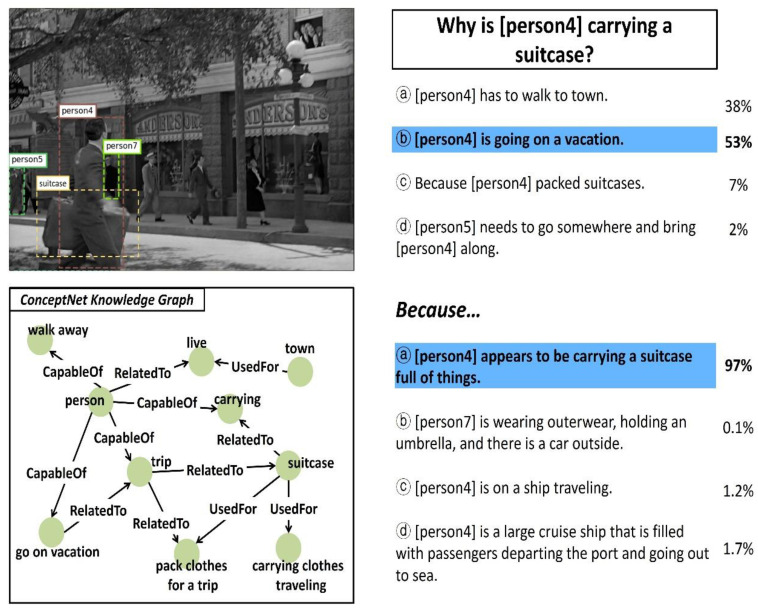
The second example of VCR task performed by the proposed model.

**Figure 11:**
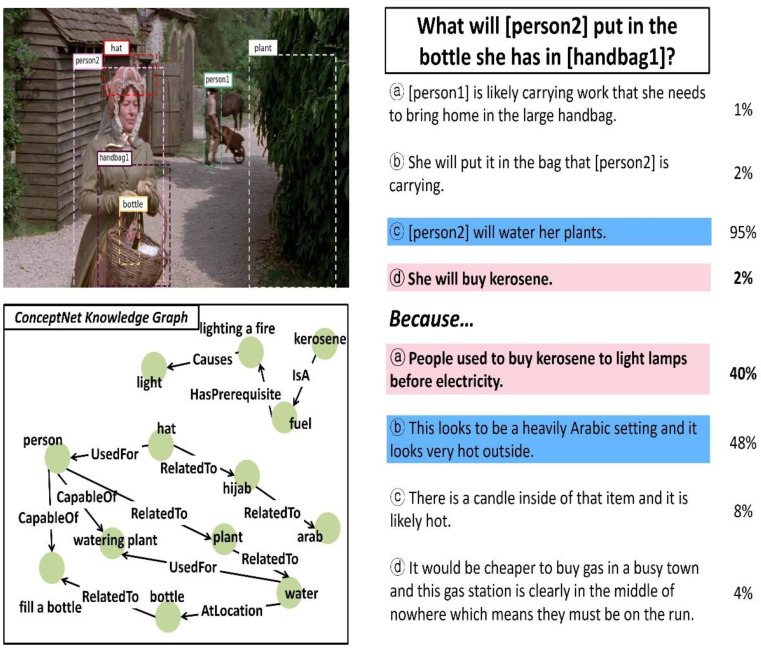
The third example of VCR task performed by the proposed model.

In this study, the performance of the ViLaKC model was qualitatively analyzed based on typical VCR tasks. Figure 9, Figure 10 and Figure 11 show some examples of VCR tasks, having Q → A and QA → R type questions and predicted answers. The upper left part shows the input image with markings of the detected object regions, and lower left part shows a portion of the related knowledge graph extracted from ConceptNet. Furthermore, the upper right part shows the candidate responses, ground truth answer, predicted answer, and confidence of prediction for the Q → A type question, and the lower right part shows them for the QA → R type question. The answers predicted by the ViLaKC model are highlighted in blue. If the predicted answer does not match the ground truth, the ground truth is highlighted in red.

The Q → A question shown in the upper part of Figure 9 is an example in which the answer can be easily predicted. “What is [person1] doing now?” is properly matched with the region of [person 1] detected in the input image. Therefore, the ViLaKC model chose the ground truth answer ⓒ with a high confidence level of 98%. For QA → R question asking about the rationale/reason in the lower part of Figure 9, the ground truth answer can be easily predicted based on simple facts that the bench mentioned only in ⓓ among the given candidate responses appears in the input image, and the objects mentioned in the rest of candidate responses are not detected in the input image. Therefore, the proposed model, which has been trained on contextualized embeddings between the input image and natural language texts, can easily select the ground truth answers for the Q → A and QA → R questions without relying on the related knowledge extracted from ConceptNet.

In contrast, the answer to the question “Why is [person4] carrying a suitcase?” in Figure 10 cannot be easily obtained by simply aligning the [person4] and [suitcase] appearing in the image with the natural language question. Therefore, basic commonsense knowledge about the entity, activity, and relationships appearing in the input data is required in addition to the input image, natural language question, and candidate responses. The ViLaKC model chose ⓑ as the answer with a 53% confidence level by effectively using the possible relationships between the [person4] and [suitcase] appearing in the natural language question and an activity [vacation] mentioned in a candidate response ⓑ within the relevant knowledge graph extracted from ConceptNet. Meanwhile, ViLaKC model also predicted that the possibility of another candidate response ⓐ being the ground truth answer was 38% based on the detected person and object and the estimated place in the input image. For the QA → R question asking about the rationale/reason in Figure 10, the ViLaKC model chose ⓐ among the candidate responses with a high confidence level of 97%. The rationale could be as follows: the fact that the [person4] is capable of [carrying] a [suitcase], as expressed in ⓐ, is identified through the relevant knowledge graph, whereas the remaining candidate responses ⓑ, ⓒ, and ⓓall mention the objects or places such as umbrella, car, ship, and port that cannot be found in the input image or the relevant knowledge graph.

Finally, in the example in Figure 11, an answer has to be provided based on the input image containing various objects for a natural language question, “What will [person2] put in the bottle she has in [handbag1]?”, which asks about an event that will occur in the future. The ground truth answer to this question is ⓓ among the candidate responses, but the ViLaKC model chose ⓒ, an incorrect answer, with a high confidence level of 95%. This is because there is a lack of basic knowledge that assures a likelihood of the [person2] [buying] [kerosene] as mentioned in ⓓ whereas there is a lot of knowledge in the relevant knowledge graph extracted from the ConceptNet, which supports the likelihood of [person2] [watering] a [plant] as mentioned in ⓒ. Furthermore, for the QA → R question of Figure 11, which asked about the rationale/reason, the ViLaKC chose ⓑincorrectly with a relatively low confidence level of 48% instead of the ground truth ⓐ, partially due to the relevant knowledge graph extracted from the ConceptNet. In other words, the ViLaKC model chose an incorrect answer ⓑdespite its low confidence level because the knowledge graph provides relevant knowledge to link the [hat] detected in the input image to the [Arabic setting] mentioned in ⓑ,whereas it does not include knowledge used to guess the relationship between the [kerosene] and [lighting lamp] mentioned in ⓐ. Through a qualitative evaluation using these typical examples, we confirmed the high performance of the ViLaKC model, which can extract question-related knowledge from an external knowledge base and use it effectively. Furthermore, as confirmed in the example of Figure 11, the performance of the ViLaKC model needs to obtain wider and richer knowledge to answer highly difficult commonsense reasoning questions.

## 5. Conclusions

This paper proposes a novel ViLaKC model for VCR [2] tasks. The model effectively extracts the knowledge relevant to the given VCR task from an external knowledge base, ConceptNet, and use it to answer the question. The ViLaKC model independently embeds the knowledge graph extracted from ConceptNet using a graph convolutional network (GCN) layer before concatenating with the image, natural language question, and candidate responses. Furthermore, the ViLaKC model uses the pretrained self-attention-based VLKEM to co-embed three different modal data, such as image, natural language question, and knowledge, into a single multimodal feature vector. Furthermore, the VLKEM module is pretrained in two stages before being used in the ViLaKC model. Particularly, in the second pretraining stage using the VCR v1.0 dataset, the ability of the VLKEM module for the contextual embeddings between multimodal data is trained using tasks such as MLMIK, MOCTK, and VLKM. The excellence of the proposed model was confirmed through various experiments using the VCR v1.0 benchmark dataset. In this study, we also found that recent pretrained multi-head self-attention models such as VisualBERT, ViLBERT, and Unicoder-VL perform better than conventional RNN-based models such as R2C, HGL, and TAB-VCR. The promising results rely on the capability of the pretrained multi-head self-attention models to learn task-agnostic joint representation of image content and natural language. However, none of the existing multi-head self-attention models have utilized commonsense knowledge in visual commonsense reasoning, which we believe will be greatly helpful in the VCR task. 

Considering the delay time of the internet and real-time requirements of VCR tasks, the ViLaKC model prefetches the relevant knowledge from ConceptNet and uses it in the VCR task after loading it on a local database. Therefore, VCR tasks can be affected by the ConceptNet knowledge base and coverage of the prefetched relevant knowledge graphs. If an appropriate knowledge graph required in the question and candidate responses has not been prefetched successfully, an incorrect answer may be chosen. Therefore, a follow-up study should be conducted to expand the relevant knowledge coverage without significantly reducing computational efficiency. Further, although the VLKEM employed in the ViLaKC model was developed for VCR tasks, it can be applied to various downstream tasks such as visual question-answering (VQA), visual dialog (VD), and vision-and-language navigation (VLN), in which vision, language, and knowledge should be used together. Therefore, a follow-up study will be conducted to expand and generalize the VLKEM for use in other applications.

## Figures and Tables

**Table 1:** Comparison between knowledge graph embedding methods.

Methods	Q → A	QA → R	Q → AR
AttentionWalk [28]	70.2	71.7	50.4
Node2vec [27]	70.8	72.8	51.4
DeepWalk [26]	71.0	73.1	52.2
GCN	72.8	75.0	54.9

**Table 2:** Comparison between the multimodal embedding methods.

Methods	Q → A	QA → R	Q → AR
Early Fusion (EF)	61.1	61.6	37.7
Late Fusion (LF)	71.6	73.6	52.8
Concurrent Fusion (CF)	72.8	75.0	54.9

**Table 3:** Comparison between the pretraining tasks.

Pretraining Tasks	Q → A	QA → R	Q → AR
MLMIK	71.1	73.3	52.3
MOCTK	61.4	65.9	40.8
VLKM	24.9	24.9	6.3
MLMIK+MOCTK	70.7	72.3	51.2
MLMIK+VLKM	72.1	74.4	53.6
MOCTK+VLKM	71.5	72.3	51.9
MLMIK+MOCTK+VLKM	72.8	75.0	54.9

**Table 4:** Comparison with existing models.

Models	Q → A	QA → R	Q → AR
Chance	0.250	0.250	0.062
R2C [2]	63.8	67.2	43.1
HGL [13]	69.4	70.6	49.1
TAB-VCR [12]	69.9	72.2	50.6
VisualBERT [15]	70.8	73.2	52.2
ViLBERT [14]	72.4	74.5	54.0
Unicoder-VL [16]	72.6	74.5	54.4
ViLaKC (Ours)	72.8	75.0	54.9

## Data Availability

The datasets used and/or analyzed during the current study are available from the corresponding author upon reasonable request.
